# Molecular mimicry between parasites and cancer: a novel approach for developing cancer vaccines and therapeutic antibodies

**DOI:** 10.1007/s00262-025-04069-1

**Published:** 2025-05-22

**Authors:** Maha Mohamed Eissa, Sonia Rifaat Ahmed Allam, Cherine Adel Ismail, Rasha Abdelmawla Ghazala, Nahla El Skhawy, Eman Ibrahim El-said Ibrahim

**Affiliations:** 1 Department of Medical Parasitology, Faculty of Medicine, Alexandria University, Al-Moassat Medical Campus, Alexandria, Egypt; 2 Department of Clinical Pharmacology, Faculty of Medicine, Alexandria University, Alexandria, Egypt; 3 Department of Medical Biochemistry, Faculty of Medicine, Alexandria University, Alexandria, Egypt

**Keywords:** Parasites, Cancer, Molecular mimicry, Cancer therapy

## Abstract

Cancer is one of the most dreaded diseases worldwide. Conventional treatments such as surgery, chemotherapy, and radiotherapy have limitations and adverse effects. Cancer immunotherapy and targeted therapies offer new treatment options. Parasite-based cancer therapy shows promise in fighting tumors. Some parasites have anti-cancer properties through multi-mechanistic strategies, with the molecular mimicry theory as a leading explanation for parasites’ anti-cancer effects. This study aimed to explore the existence of shared antigenic proteins between parasites (*Trichinella spiralis*, *Schistosoma mansoni*, and *Toxoplasma gondii*) and cancer cell lines (MCF-7 human breast cancer and A549 human lung cancer). Polyclonal antisera against *T. spiralis*, *S. mansoni*, and *T. gondii* parasites were generated in rabbits. Antibody reactivity with extracts of MCF-7 and A549 cancer cells was detected using SDS-PAGE and immunoblotting. Results documented the molecular mimicry between parasites and cancers as it revealed cross-reactive bands when using *T. spiralis* antibodies against MCF-7 and A549 cancer cell extracts at approximate molecular weights of 70 and 35 kDa, and with *S. mansoni* antibodies at an approximate molecular weight of 80 kDa. *Toxoplasma gondii* antibodies neither reacted with MCF-7 human breast cancer nor A549 human lung cancer cell extracts. Results of this study could establish a foundation for subsequent investigation among a broad range of parasites for molecular mimicry with cancers. Identification, molecular characterization, and investigation of the anti-neoplastic activity of these cross-reactive antigens could shed light on new pathways for the potential development of a novel class of innovative cancer vaccine candidates and therapeutic antibodies of parasitic origin for cancer immunotherapy and targeted therapy.

## Introduction

Cancer remains a major global health concern, despite global efforts in diagnosis and treatment. Ahead of World Cancer Day, the World Health Organization (WHO) in 2024 highlighted the growing global cancer burden. Lung cancer is the most commonly occurring cancer worldwide with 2.5 million new cases accounting for 12.4% of the total new cases. Regardless of the subtypes, it is considered the deadliest cancer with a 22% five-year survival rate. The second most common cancer is breast cancer with 2.3 million cases accounting for 11.6% of the total new cases. Breast cancer is highly heterogeneous in its pathological characteristics, with some cases showing a slow tumor growth rate with an excellent prognosis, while others are aggressive tumors [1].

Surgery, radiotherapy, and chemotherapy are the most recommended and widely used policies in cancer treatment. Despite dramatic advances in conventional cancer policies, their application has significant adverse effects, and cost-effective concerns with limitations depending on tumor grade, stage, and patient tolerance [2]. Chemotherapeutic drugs and radiation therapies also have various impacts on patients' cognitive function which is referred to as chemo-brain. In addition, chemoresistance continues to be a major problem in cancer therapy and is responsible for most relapses and poor survival outcomes in patients [2]. Therefore, cancer research has focused on exploring innovative cancer treatments beyond these conventional methods.

Immunotherapy and targeted therapy represent rapidly advancing therapies widely appreciated as groundbreaking in cancer treatment. Immunotherapy boosts patients’ immune response to fight cancer subsequently targeting and vigorously attacking cancer cells [3]. Meanwhile, targeted therapy seeks to selectively affect cancer cells or the tumor microenvironment that promotes cancer growth. It focuses on specific cancerous molecules that are critical via suppressing cell migration, differentiation, and proliferation. Therefore, targeted therapy offers to minimize the off-target adverse effects [4].

The main characteristics of cancer cells are their ability to deceive the immune system and create an immune-resistance tumor microenvironment. The defining hallmark of cancer cells is the expression of two main categories of antigens: tumor-associated antigens (TAAs) and tumor-specific antigens (TSAs). Tumor-associated antigens are overexpressed self-antigens and composed of differentiation antigens such as carcinoembryonic antigen, mucin-1 which is hypoglycosylated in adenocarcinomas, carbohydrate antigens (N-acetylgalactosamine O-serine/threonine (Tn), sialyl-Tn (STn), thymidine kinase (Tk) and Thomsen Friedenreich (TF)), *p53* suppressor gene and heat shock protein (HSP). Tumor-associated antigens have important roles in metastasis, cell adhesion, and invasion [5]. Meanwhile, TSAs are strictly specific to tumor cells and responsible for cancer mutation. They arise mostly from oncogenic driver mutations that generate novel peptide sequences [6].

The application of tumor-expressed antigens has emerged into the scientific consciousness for cancer immune therapy. However, autologous cancer vaccination strategies are insufficient to elicit a broad immune response. In addition, there is immune tolerance to self-antigens from cancer cells. These obstacles could be overcome by using xenogeneic orthologues antigens such as pathogens’ antigens [7]. Pathogens, being foreign entities, can trigger a robust immune response, making them potential candidates for immunotherapy trials [8, 9].

The relationship between parasites and cancer has been a subject of scientific interest. Some parasites were confirmed as cancer inducers such *as Schistosoma haematobium* (*S. haematobium*), *Clonorchis sinensis* (*C. sinensis*), and *Opisthorchis viverrini* (*O. viverrini*). However, a negative correlation between other parasites and some cancer types has been documented [9].

Parasite-based cancer therapy is dependent on multi-faceted mechanisms including induction of apoptosis, inhibition of angiogenesis, immunomodulation of tumor microenvironment, and molecular mimicry theory [10, 11]

The concept of shared cross-reactive antigens between parasites and cancer cells (molecular mimicry theory) glows as a sparkling concept that sounds noteworthy and deserves further investigation. Parasite-derived antigens, mainly glycoproteins, are highly immunogenic and reveal a high degree of homology with cancer antigens. For example, Tn antigen is expressed by different parasites such as *Schistosoma mansoni* (*S. mansoni*) and schistosomula, *Echinococcus granulosus* (*E. granulosus*) and its larval stage, and *Trypanosoma cruzi* (*T. cruzi*) [12–14]. Tk antigen is found in *Taenia crassiceps*, *Mesocestoides vogae* and *Taenia hydatigena* [15]. Meanwhile, TF antigen is expressed by *Fasciola hepatica*, *S. mansoni*, and *E. granulosus* [16–18]. STn antigen is expressed by *E. granulosus* and *T. cruzi* [13, 14]. Surprisingly, *T. cruzi* antigens displayed common epitopes with mammalian mucins [19]. In addition, N and O-linked glycans and the enzyme required for mucin-type O-glycosylation have been reported in *Toxoplasma gondii* (*T. gondii*) tachyzoites [20, 21].

Multiple experimental studies using parasites, their derived molecules and antisera demonstrated promising anti-cancerous activities against multiple cancer types. For instance, *Plasmodium* [22–24], *T. gondii* [25, 26], *Trypanosoma* species (spp.) [27, 28], *Trichomonas vaginalis* (*T. vaginalis*) [29], *E. granulosus* [30, 31], and *Trichinella spiralis* (*T. spiralis*) [32, 33] have reported potent anti-cancerous efficacy against lung cancer.

Regarding breast cancer, multiple research studies have explored the role of parasites in breast cancer therapy. For example, *Plasmodium* spp., [34] *T. gondii*, [35–37] *Neospora caninum* (*N. caninum*) [38], *Besnoitia jellisoni* (*B. jellisoni*) [39], *T. cruzi* [40, 41], and *Leishmania* [42] are among the suggested protozoa in treating breast cancer. Meanwhile, *E. granulosus* [43–45], *Taenia solium* [46], and *T. spiralis* [47] are examples of helminths that have been reported to have anti-neoplastic effects against breast cancer. Interestingly, *S. mansoni*, which expresses human cancer-associated antigens Tn and TF, was recently reported by the current authors to exhibit anti-neoplastic activity against breast cancer in a pre-clinical study [48].

In this study, our goal was to explore molecular mimicry between parasites and cancers by investigating the presence of cross-reactive antigens between parasites (*T. spiralis*, *S. mansoni*, and *T. gondii*) and cancer cells (MCF-7 human breast and A549 human lung cancer cell lines.

## Materials and methods

### Maintenance of parasites life cycles and antigens preparations

Parasitic antigens were prepared from the infective stage of the following parasites: *T. spiralis* (larvae), *S. mansoni* (cercariae), and *T. gondii* (tachyzoites). The life cycles of these parasites were maintained at the laboratory of the Medical Parasitology Department, Faculty of Medicine, Alexandria University, Egypt.

### Autoclaved *Trichinella spiralis* larval antigen (A*Ts*A)

*Trichinella spiralis* life cycle was maintained by serial passages in adult Wistar rats. The larvae were collected from infected rats by the digestion method and were washed five times in phosphate buffer saline (PBS) at 1000 rpm for 10 min [49]. The final pellet was resuspended in PBS into a screw-capped vial, autoclaved under the pressure of 15 Ib at 121 °C for 15 min, and stored at − 20 °C until later use [50].

### Autoclaved *Schistosoma mansoni* cercarial antigen (A*Sm*A)

*Schistosoma mansoni* life cycle was maintained by passage through *Biomphalaria alexandrina* snails and Swiss albino mice [51]. *S. mansoni* cercariae were shed from snails, gravity-sedimented for two hours at 4 °C, excess fluid removed, and the pellet was resuspended in PBS into a screw-capped vial, autoclaved under the pressure of 15 Ib at 121 °C for 15 min, and stored at − 20 °C until later use [52].

### Autoclaved *Toxoplasma gondii* tachyzoites antigen (A*Tg*A)

*Toxoplasma gondii* (virulent RH HXGPRT (−) strain) life cycle was maintained through serial intraperitoneal passages of tachyzoites in Swiss albino mice [53]. The freshly collected tachyzoites were centrifuged at 500 rpm for five minutes to allow sedimentation of leukocytes and heavier particles. The supernatant was then collected and washed three times at 2000 rpm for five minutes. The final pellet was resuspended in PBS into a screw-capped vial, autoclaved under the pressure of 15 Ib at 121 °C for 15 min, and stored at − 20 °C until later use [37].

### Preparation of hyperimmune antisera

#### Preparation of antisera against A*Ts*A

Male New Zealand albino rabbit (2.5 kg) was immunized by intramuscular injection of 300 μg of A*Ts*A emulsified in Freund's complete adjuvant (FCA). Two booster doses of the antigen emulsified in an equal volume of FCA were injected subcutaneously at two-week intervals. Blood samples were collected four days after the last injection. The serum was separated from the collected blood via centrifugation at 2000 rpm for 15 min then stored at -20 °C for later use [54].

#### Preparation of antisera against A*Sm*A

Male New Zealand albino rabbit (2.5 kg) was immunized with 5 mg of A*Sm*A in FCA. The antigen was emulsified in an equal volume of adjuvant and the rabbit was injected intramuscularly once a week for six weeks. Blood samples were collected one week after the last injection. The serum was separated from the collected blood via centrifugation at 2000 rpm for 15 min, then stored at − 20 °C for later use [55, 56].

#### Preparation of antisera against A*Tg*A

Male New Zealand albino rabbit (2.5 kg) was immunized by subcutaneous injection of 0.5 mg of A*Tg*A emulsified in an equal volume of FCA. Three booster doses of the antigen emulsified in an equal volume of FCA were injected; the first dose was injected two weeks after the priming dose. In the meantime, the second and third booster doses were injected after one week with a one-week interval. Blood samples were collected one week after the last injection. The serum was separated from the collected blood via centrifugation at 2000 rpm for 15 min, then stored at − 20 °C for later use [37].

For confirmation of the positivity of parasite IgG antibodies in rabbits’ antisera, an enzyme-linked immunoassay (ELISA) reaction was performed separately for each antiserum using its corresponding antigen. In brief, ninety-six-well plates were coated with the parasitic antigens diluted 1:20 with carbonate buffer (100 μl/ well) (A*Sm*A, A*Ts*A and A*Tg*A). Following overnight incubation at 4 °C, blocking buffer (1% bovine albumin) was added to the plates (200 μl/ well) for two hours at room temperature, then washed with sodium chloride buffer containing 0.05% Tween 20. One hundred μl of the prepared antisera diluted in blocking buffer was added to its corresponding antigen and incubated at 37 °C for two hours. The plates were then washed four times with PBS. One hundred μl of diluted secondary antibody (goat anti-rabbit; Sigma-Aldrich) in blocking buffer was added and incubated at 37 °C for one hour. Finally, following washing, the plates were incubated with the chromogenic substrate and the optical densities of the wells were read at 450 nm using an ELISA reader (BIO-RAD). Serum from a non-immunized rabbit was used as a negative control [57, 58].

#### Preparation of cancer cell line lysates

MCF-7 human breast cancer and A549 lung cancer cell pellets (7 × 10^6^ cells) were purchased from the Center of Excellence for Research in Regenerative Medicine and its Applications, Faculty of Medicine, Alexandria University. MCF-7 and A549 cell pellets were washed twice with PBS and resuspended in modified radioimmunoprecipitation assay buffer (RIPA) lysis and extraction buffer containing a cocktail of protease inhibitors (Thermo Fisher Scientific) for 15 min on ice. Cell extracts were centrifuged for 10 min at 14,000 rpm to pellet cell debris. Supernatants were collected and stored at − 20 °C until later use [59, 60].

### Comparative immunoproteomic analysis between autoclaved parasitic antigens and extracts of MCF-7 and A549 cancer cells

#### One-dimensional sodium dodecyl polyacrylamide gel electrophoresis (SDS-PAGE)

Sodium dodecyl polyacrylamide gel electrophoresis was conducted for extracts of MCF-7, A549 cancer cells, and parasitic antigens A*Ts*A, A*Sm*A, and A*Tg*A. The protein concentration of each sample was assessed using a NanoDrop™ 2000 spectrophotometer and was expressed in mg/ml. BLUeye Prestained Protein Ladder (BIO-HELIX) and samples were allowed to run using an 8% stacking gel followed by 12% resolving gel (Tris–HCl PH:8.8). Gel was stained with Commasie Brilliant Blue R 250 (Sigma-Aldrich), documented using Gel Doc™ XR + (BIO-RAD) documentation system and analyzed by Image Lab software 5.1 (BIO-RAD) [61].

#### Immunoblotting and immunodetection

Three separate SDS-PAGE gels; MCF-7, A549 and A*Ts*A; MCF-7, A549 and A*Tg*A and MCF-7, A549 and A*Sm*A were electrophoresed. Then, each SDS-PAGE-separated proteins were transferred to nitrocellulose membranes and probed with the corresponding rabbit antisera diluted 1:100 in Tris-buffered saline with 0.5% v/v Tween 20 (TBST) overnight at 4 °C as the primary antibody, followed by two hours at room temperature in a solution of horseradish peroxidase (HRP)-conjugated goat anti-rabbit IgG antibodies (Sigma-Aldrich) diluted 1:1000 in TBST as the secondary antibody. Finally, it was washed, and chemiluminescent detection was performed. It was documented using Gel Doc™ XR + (BIO-RAD) documentation system and analyzed by Image Lab software 5.1 (BIO-RAD) [62]. At least three replicates of SDS-PAGE gels and immunoblots have been done for cross-reactive experiments.

## Results

### SDS-PAGE analysis

**Fig. 1:**
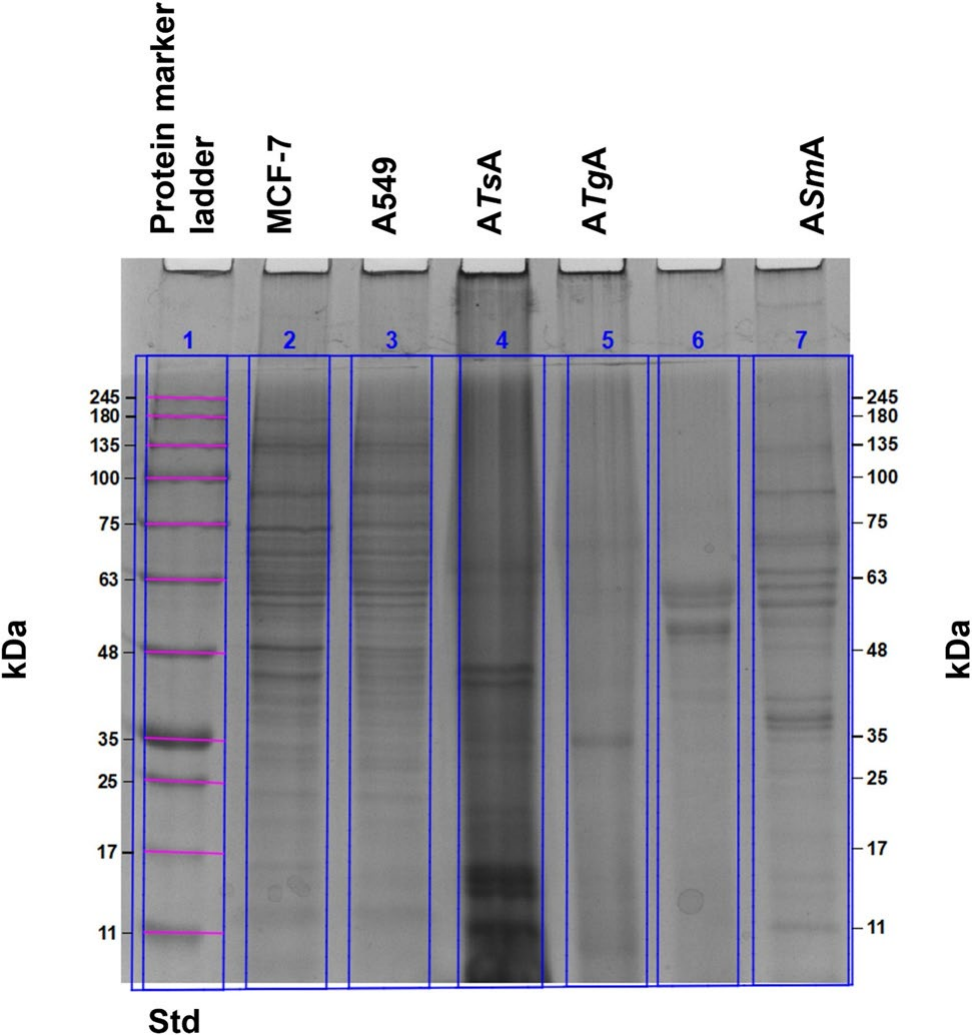
Sodium dodecyl polyacrylamide gel electrophoresis of cell extracts of MCF-7 human breast cancer, A549 human lung cancer and parasitic antigens (A*Ts*A, A*Tg*A and A*Sm*A). Lane 1: BLUeye Prestained Protein Ladder electrophoretic pattern, lane 2: MCF-7 human breast cancer cell extracts electrophoretic pattern, lane 3: A549 human lung cancer cell extract electrophoretic pattern, lane 4: A*Ts*A electrophoretic pattern, lane 5: A*Tg*A electrophoretic pattern, lane 6: electrophoretic pattern of external antigen and lane 7: A*Sm*A electrophoretic pattern. A*Ts*A: autoclaved *T. spiralis* antigen; A*Tg*A: autoclaved *T. gondii* antigen; A*Sm*A: autoclaved *S. mansoni* antigen. Std: Standard

To test the molecular mimicry theory, SDS-PAGE was conducted. Figure 1 and Table 1 demonstrate the data analysis for the SDS-PAGE lanes profile showing molecular weight ranges for cell extracts of MCF-7 human breast cancer, A549 human lung cancer, and parasitic antigens (A*Ts*A, A*Tg*A, and A*Sm*A). For MCF-7 human breast cancer, the molecular weights of protein bands ranged approximately from 180 to 11 kDa (Fig. 1, Lane 2); meanwhile, for A549 human lung cancer, the molecular weights of protein bands ranged from approximately 200–11 kDa (Fig. 1, Lane 3). As regards parasitic antigens, the molecular weights of protein bands of A*Ts*A ranged from approximately 75–11 kDa (Fig. 1, Lane 4), of A*Tg*A ranged from approximately 70–11 kDa (Fig. 1, Lane 5), and of A*Sm*A ranged from approximately 135–12 kDa (Fig. 1, Lane 7).

**Table 1:** Data analysis for SDS-PAGE lanes profile showing molecular weight ranges for cells extracts of MCF-7 human breast cancer cell extracts, A549 human lung cancer cell extracts, and parasitic antigens (A*Ts*A, A*Tg*A, and A*Sm*A)

Lanes	Molecular weights range in kDa
Lane 1: Standard protein marker	245–11
Lane 2: MCF-7 cell extracts	180–11
Lane 3: A549 cell extracts	200–11
Lane 4: A*Ts*A	75–11
Lane 5: A*Tg*A	70–11
Lane 7: A*Sm*A	135–12

### Immunoblotting detection

**Fig. 2:**
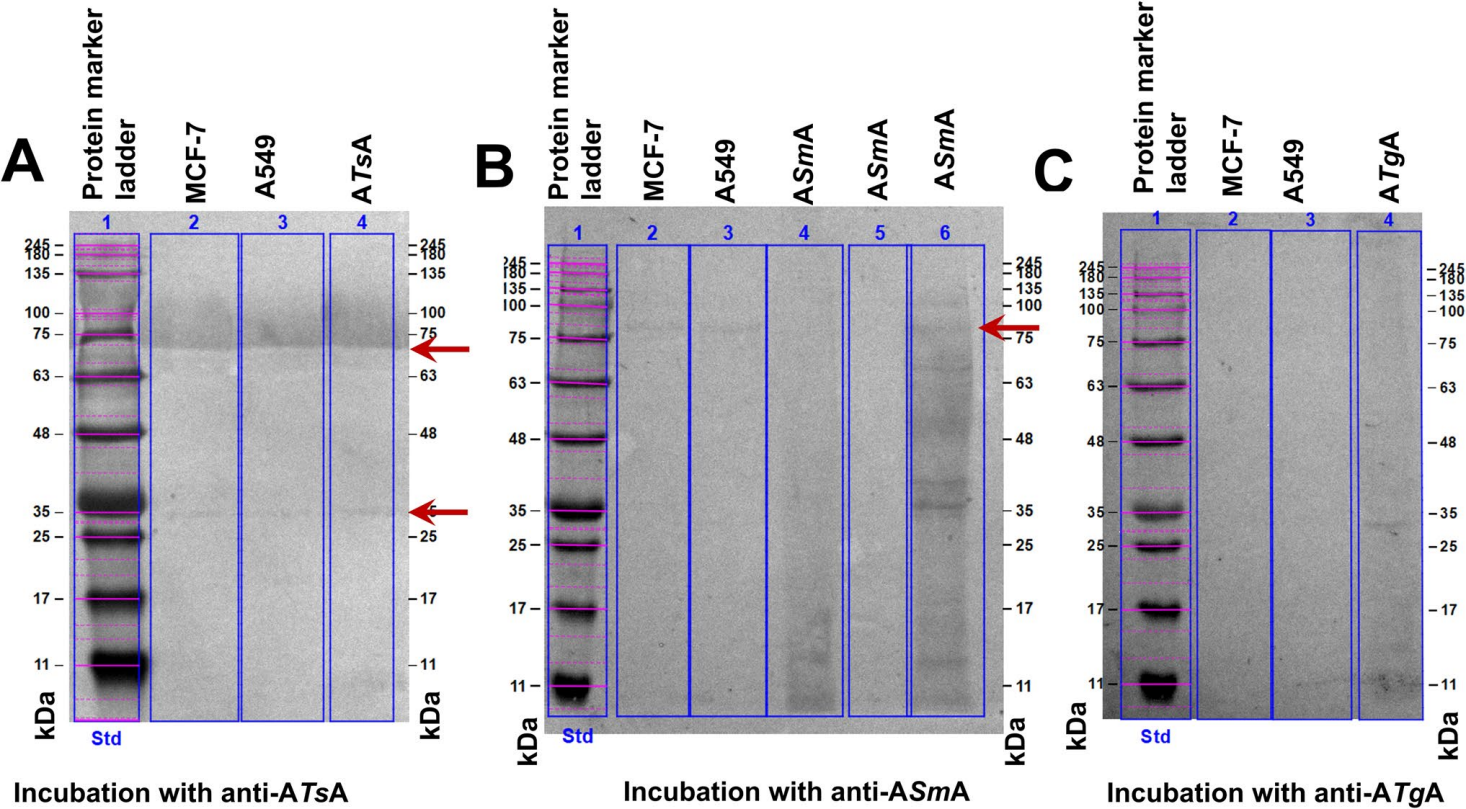
Immunoblotting analysis of cell extracts of MCF-7 human breast cancer, A549 human lung cancer and parasitic antigens (A*Ts*A, A*Tg*A and A*Sm*A). **A** After incubation with anti-A*Ts*A antibodies; lane 1: protein molecular weight marker, lane 2: MCF-7 human breast cancer cell extract, lane 3: A549 human lung cancer cell extract and lane 4: A*Ts*A. **B** After incubation with anti-A*Sm*A antibodies; lane 1: protein molecular weight marker, lane 2: MCF-7 human breast cancer cell extract, lane 3: A549 human lung cancer cell extract and lanes 4, 5 & 6: A*Sm*A. **C** After incubation with anti-A*Tg*A antibodies; lane 1: protein molecular weight marker, lane 2: MCF-7 human breast cancer cell extract, lane 3: A549 human lung cancer cell extract and lane 4: A*Tg*A. Red arrow points out the shared cross-reactive bands. A*Ts*A: autoclaved *T. spiralis* antigen; A*Tg*A: autoclaved *T. gondii* antigen; A*Sm*A: autoclaved *S. mansoni* antigen. Std: Standard

The reactivity of prepared polyclonal sera was confirmed using ELISA. Immunoblotting analysis was performed to verify the presence of cross-reactive antigens between cell extracts of MCF-7 and A549 cancer cells and parasitic antigens against their corresponding polyclonal antibodies. Upon incubation with anti-*T. spiralis* antibodies, two prominent bands corresponding to approximately 70 and 35 kDa were consistently detected in A*Ts*A and both MCF-7 human breast cancer and A549 human lung cancer cell extracts (Fig. 2A). When applying anti-*S. mansoni* antibodies, a prominent band corresponding to approximately 80 kDa was consistently detected in A*Sm*A and both MCF-7 human breast cancer and A549 human lung cancer cell extracts (Fig. 2B). Interestingly, when using anti-*T. gondii* antibodies, no cross-reactive bands were detected in both MCF-7 human breast cancer and A549 human lung cancer cell extracts (Fig. 2C). However, only two reactive bands were detected in A*Tg*A at approximately 30 and 13 kDa.

Based on the aforementioned results, the presence of cross-reactive antigens between MCF-7 human breast cancer and A549 human lung cancer cell extracts was detected after incubation with anti-*T. spiralis* antibodies at approximately molecular weights 70 and 35 kDa and anti-*S. mansoni* at 80 kDa. Meanwhile, no cross-reactive antigens were detected after incubation with anti-*T. gondii* antibodies.

## Discussion

Cytotoxic agent-based chemotherapy has been the main strategy for the treatment of a wide range of cancers for decades. Most of these chemotherapeutic agents show severe adverse effects due to a non-specific impact on normal healthy cells. To address this issue, immunotherapy and targeted therapy have significantly advanced cancer therapeutic strategies [63].

Cancer immunotherapy encompasses several promising strategies such as cancer vaccines, antibody-based therapies, adoptive cell transfer, viral-based therapies, checkpoint inhibitors, and cytokines [64]. While monoclonal antibodies and small molecule inhibitors are common targeted therapies [4]. For example, trastuzumab, a monoclonal antibody targeting human epidermal growth factor receptor-2 (HER-2) is approved by the Food and Drug Administration (FDA) as a first-line treatment of HER-2-positive cancer [65]. Similarly, pembrolizumab, an FDA-approved monoclonal antibody targets programmed cell death-1 (PD-1) protein and is effective against breast, lung, gastric, and melanoma cancers [66]. Antibody–drug conjugates and bispecific antibodies are innovative approaches in antibody-based cancer therapies [67, 68]. In addition, bortezomib, a proteasome inhibitor, has been approved for treating multiple myeloma and mantle cell lymphoma [69].

Parasite-based cancer therapeutics hold considerable promising implications in the oncology field. Parasites can be utilized for cancer immunotherapy by providing highly immunogenic cancer vaccine candidates [42, 70, 71], acting as immunomodulators [72, 73], adjuvants [24, 74], and inhibitors of nuclear factor kappa B (NF-κB) [26]. Additionally, they could be used as oncolytic agents [75] and for developing therapeutic monoclonal/polyclonal antibodies [33, 76, 77], for targeted cancer therapy, as they can selectively attack cancer cells, leading to their destruction through antibody-dependent cell cytotoxicity (ADCC) while sparing healthy ones [78].

The molecular mimicry theory, among others, has been postulated to be the chief mechanism behind parasites’ anti-neoplastic activity. This was verified by the unique selectivity of certain parasites against specific types of cancers. For instance, Eissa et al. (2019) demonstrated that although both *S. mansoni* and *T. spiralis* antigens induced potent immunomodulatory potential in a murine model of colon cancer, yet anti-neoplastic activity was only demonstrated for *S. mansoni* [52]. This was explained by the shared antigens between the *Schistosoma* parasite and cancer, as human cancer-associated antigens Tn and TF were reported to be expressed by *S. mansoni* parasite and its schistosomula [12, 17]. Such findings have highlighted the molecular mimicry theory between parasites and cancer cells as a research hotspot. In this view, the present study investigated the presence of molecular mimicry and cross-reactive antigens between MCF-7 human breast and A549 lung cancer cells and parasites like *T. spiralis*, *T. gondii* and *S. mansoni*.

In the current study, immunoproteomic profiling showed different specific bands for each parasite and each cancer cell. Hyperimmune sera from immunized rabbits against each parasite were used to detect cross-reactivity between each parasite’s antigen and each cancer cell extract. Results showed that MCF-7 and A549 cancer cell extracts shared two antigens with A*Ts*A at molecular weights of approximately 70 and 35 kDa. In alignment, several studies have demonstrated cross-reactive antigens between *T. spiralis* and various cancer types, including Lewis lung (LL) cancer, myeloma, and osteosarcoma [79–81].

For example, a fully human single-chain antibody (ScFv) raised against *T. spiralis* 7 trans-membrane receptors (Ts7TMR) bound specifically to A549 lung cancer cells [82]. Furthermore, this antibody inhibited lung cancer growth in both *in vitro* and *in vivo* experiments [33]. *Trichinella spiralis* small heat shock protein (sHSP-DQ 986457) was identified as a gene associated with LL cancer, demonstrating cross-reactivity with LL cancer antisera [81].

In another study, a 33 kDa *T. spiralis* antigen showed a reaction with anti- Sp2/0 myeloma cells, with tropomyosin being a significant component of myeloma-associated antigens. Immunization with tropomyosin and crude *T. spiralis* antigen resulted in a similar antitumor effect in *in vivo* testing indicating its important role in eliciting cross-protective immunity [79]. Furthermore, a *T. spiralis* cDNA expression library demonstrated cross-reactivity with Sp2/0 myeloma antisera at approximately 15.6 kDa, the TS2 antigen gene, with six predicted cross-reactive epitopes [80]. In the SP2/0 myeloma mice model infected with *T. spiralis,* genes encoding RpL41, NKTR, Rbbp4, and ANXA2 were enriched in tumor cells, suggesting a potential role in tumor growth inhibition [83].

Additionally, cross-reactive antigens were detected between *T. spiralis* and osteosarcoma, where seven cross-antigen genes between cDNA expression library of muscle larvae and anti-MG-63 osteosarcoma cells antisera were found. Among these antigens, (X/7M_003375331.1) which encodes tumor protein D52 (TPD52), was found to have the highest hydrophilicity with a predicted score of 0.7106 indicating that this protein may be the most protective among the seven detected genes. Anti-TPD52 demonstrated significant anti-cancer activity against osteosarcoma in both *in vitro* and *in vivo* experiments, outperforming anti-*T. spiralis* antiserum in boosting immunity without causing histopathological damage [77].

In the present study, anti-A*Sm*A antibodies cross-reacted with both MCF-7 human breast and A549 cancer cell extracts at a molecular weight of approximately 80 kDa. Previous research in 2006 showed that sera from *S. mansoni* infected mice reacted with human gastric adenocarcinoma and bladder carcinoma cell lines [17]. To the best of our knowledge, this is the first report to demonstrate shared antigens between *S. mansoni* and cancer cells. These findings support the anti-neoplastic activity of A*Sm*A against breast cancer in a pre-clinical study [48] and other types of cancer such as colon cancer [52], sarcoma, [84] and histiocytoma [85]. This suggests the potential of *S. mansoni* antigens as a promising candidate for cancer immunotherapy.

*Toxoplasma gondii* has been the focus of many experimental studies and displayed potent anti-neoplastic activity against breast cancer [35, 37] and lung cancer [25, 26]. The postulated mechanisms were mainly hypothesized through their immunomodulatory mechanism, direct invasion, and cytotoxic effect on cancer cells [86]. The molecular mimicry theory has been a lightly touched topic as regards *Toxoplasma* and breast /lung cancer.

Interestingly, in the current study, anti-A*Tg*A antibodies did not react with MCF-7 human breast or human lung cancer cell extracts. Our results concerning MCF-7 human breast cancer cells align with those of El Skhawy, 2022, who demonstrated the absence of cross-reactivity between A*Tg*A and MCF-7 human breast cancer cell extracts, while in the same study, four cross-reactive bands were detected with Ehrlich carcinoma, a murine mammary carcinoma, using similar parameters to our current study at an approximate molecular weights of 60, 26, 22 and 12.5 kDa [87]. This justified the potent anti-neoplastic activity of A*Tg*A against Ehrlich solid carcinoma observed in both prophylactic and therapeutic experiments [36, 37]. Remarkably, our results for the absence of *Toxoplasma* cross-reactivity with MCF-7 breast cancer cells are controversial with those published in the literature. This may be related to the differences in experimental parameters used in the different studies such as the strain of *Toxoplasma*, the type of antigen, the source of antisera (rabbit *vs* human), the type of cancer cell line (human *vs* murine), the form of cancer cell (intact cells *vs* cell extract), and the detection technique employed. For example, Mohamadi et al. (2019) used *T. gondii* lysate antigen and the (4T1) murine cancer cell line. They postulated shared antigens between *Toxoplasma* and murine intact breast cancer cells via the binding of *Toxoplasma* antibodies on the surface of breast cancer cells using flow cytometry [58]. Additionally, Hosseini et al. (2023) used human sera from *Toxoplasma*-positive patients and examined both MCF-7 human breast cancer and 4T1 murine breast cancer cell extracts [88]. However, our results do not suggest disregarding the potential anti-neoplastic role of *Toxoplasma* as a plethora of experimental studies have demonstrated the potent anti-neoplastic activity of *Toxoplasma* in nearly all experimental cancer models investigated to date [11, 73, 74, 89]. The *Toxoplasma* parasite has been found to induce anti-cancer activity through different mechanistic strategies such as the induction of apoptosis [90–92], anti-tumor immune response [93], smart targeting of the tumor microenvironment [93–96], and anti-angiogenesis [25]. Other studies demonstrated that dense granule 16*-*derived from *T. gondii* enhanced the anti-neoplastic efficacy of irinotecan against non-small-cell lung carcinoma cells, by inhibiting the NF-қB activation [26]. Additionally, *T. gondii* profilin-like protein showed a marked auxiliary role as an adjuvant with the autologous whole-tumor-cell vaccine against colon cancer [97]. Interestingly, the combination therapy of *T. gondii*- deficient dense granule 17 and anti-PD- 1 antibody elicited a significant anti-tumor immune response with a synergic effect against melanomas [98]. In future studies, researchers should further explore the diverse mechanisms of *Toxoplasma’s* anti-neoplastic activity and consider parameter variations to gain a comprehensive understanding of its potential in cancer treatment.

In addition to our findings, several studies have reported molecular mimicry between parasites such as *E. granulosus, Setaria equina* (*S. equina*), and *T. cruzi,* and certain cancer types. *E. granulosus*, the dog tapeworm, and its derived antigens and antisera have demonstrated potent anti-neoplastic activity in several studies. The parasite and its larval stage, hydatid cyst, are rich in glycosylated antigens such as Tn, Sial Tn [13], and TF [16] antigens which are prominent TAAs. Studies have also demonstrated the anti-cancerous activity of *E. granulosus* against; breast cancer [44, 99], colorectal, [100] lung, [31] and bone cancers [101].

In breast cancer, antisera raised against hydatid cyst antigens reacted with 4T1 breast cancer cells [102–104]. Similarly, breast cancer patients’ sera showed cross-reactivity with ~ 27/28 kDa derived from hydatid cyst wall antigens [103, 105]. In addition, ~ 40 kDa bands derived from hydatid cyst fluid interacted with breast cancer patients’ sera [104, 106]. Interestingly, prophylactic immunization with this ~ 27/28 kDa protein demonstrated significant anti-neoplastic activity in a breast cancer murine model [103, 107].

In lung cancer, an earlier study in 1979 reported possible antigenic similarity between pulmonary carcinoma and cysts of *E. granulosus.* A broad and intense band was observed in an immunoelectrophoretic test between serum from a patient with pulmonary carcinoma and hydatid cyst fluid [108]. Notably, Berriel et al. (2021) supported the anti-neoplastic potential of *E. granulosus* against lung cancer. They reported that anti-hydatid cyst fluid antibodies recognized membrane and intracellular molecules in LL/2 cancer. In addition, hydatid cyst fluid immunization showed a protective effect against LL/2 lung cancer in murine model [31].

In colorectal cancer, anti-hydatid cyst fluid antibodies identified cell surface and intracellular antigens in CT26 colon cancer cells and cross-reacted with five protein spots of CT26 colon cancer proteins. These proteins were analyzed by MALDI TOF/TOF–MS, and two of them were identified as mortalin and creatine kinase M-type. Although no significant homology between creatine kinase M-type and *E. granulosus* proteins was found, interestingly, colon cancer cells (CT26) mortalin showed 60% homology with *E. granulosus* HSP-70 potentially justifying the anti-cancer properties of *E. granulosus* in a mouse model of colorectal cancer [100, 109]. In bone cancer, hydatid cyst wall antigens cross-reacted with sera from patients with bone cancers at 70 and 53 kDa [101].

*Setaria equina* is a common vector-borne parasite of equines worldwide. Adult worms are primarily found in the peritoneal cavity of horses and donkeys [110]. Abdel-Latif and Sakran (2016) explored the molecular mimicry between this nematode parasite and certain cancers. They identified cross-reactivity between anti-*Setaria equina* antibodies and cell extracts from Huh-7 hepatoma and MCF-7 human breast cancer cells at 75 and 70 kDa, potentially corresponding to Glucose-6-phosphate dehydrogenase and HSP-70, respectively [111]. These shared antigens may explain the anti-cancer effects of *S. equina* excretory–secretory products against hepatocellular carcinoma in a rat model [112, 113].

*Trypanosoma cruzi,* a parasite causing Chagas' disease in humans, has shown anti-cancer properties against various types of cancer in both *in vitro* and *in vivo* experiments [28, 40, 76, 114]. Zenina et al. (2008) demonstrated that antigens in *T. cruzi* with anti-cancer activity have common epitopes with mammalian mucins. Immunization of mice with type II and III mucins shared antigens of *T. cruzi* inhibited the Ehrlich adenocarcinoma growth and induced onco-protective effects [41]. In addition, anti- *T. cruzi* antibodies showed anti-cancerous efficacy against mammary carcinoma [41, 115]. Notably, anti-*T. cruzi* antibodies specifically recognize human colon cancer cell lines (HT29 and LS-174 T) and breast cancer cell lines (T47D and MCF-7) [40].

Remarkably, anti-*T. cruzi* antibodies have been found to recognize and cross-react with membrane and intracellular molecules in lung cancer cells. Immunization of mice bearing lung cancer with *T. cruzi* lysate showed potent reduction in tumor size and increase in survival rates [28]. Regarding hematologic cancer, polyclonal anti- *T. cruzi* antibodies cross-reacted with acute lymphoblastic leukemia SUPB15 cell line. A prominent band of approximately to 100 kDa protein was consistently detected and was identified as nucleolin protein [76]. In addition, a previous study found that polyclonal anti- *T. cruzi* antibodies species specific cross-reacted in multiple bands with acute lymphoblastic leukemia cells (95, 75, 70, 55, 50, and 43 kDa) and neuroblastoma (140, 90, 85, 80, 70, and 55 kDa) [116].

From the aforementioned data, it is evident that epitope homology and cross-reactivity between parasites and cancer cells are potent explanations for understanding the mechanisms involved in the anti-cancer activities of a wide range of parasites. Administration of parasitic antigens could flare up the immune response to eradicate parasites and simultaneously, homologous malignant cells [10]. Thereby, they could directly raise innate, humoral, and cell-mediated cytotoxic immunity against cancers; they might enable cross-reactive T cells to recognize and kill tumor cells via molecular mimicry [117]. In addition, polyclonal antibodies against these shared antigens can mediate ADCC targeting tumor cells for destruction, promotion of antigen presentation and induction of anti-tumor responses [118]. Thus, the results of this study could establish a foundation for subsequent investigation among a broad range of parasites and different types of cancer. A systematic exploration of parasites could illuminate new pathways for potentially discovering a novel class of innovative cancer vaccine candidates and therapeutic antibodies of parasitic origin, among others, for cancer therapy. Interdisciplinary collaboration among researchers from different scientific disciplines, funding agencies, the pharmaceutical industry, along with modern technology, is crucial for overcoming challenges in identifying shared antigenic components between parasites and cancers, understanding mechanisms of action, advancing to clinical trials, as well as commercial production, regulatory and logistical challenges.

## Conclusion

This study demonstrated the molecular mimicry between certain parasites and various cancer types, potentially explaining one of the underlying mechanisms of parasites’ anti-neoplastic activity. Both anti-A*Ts*A and A*Sm*A antibodies revealed cross-reactive antigens with cell extracts from MCF-7 human breast cancer and A549 lung cancer cells at different molecular weights. On the contrary, anti-A*Tg*A antibodies neither reacted with MCF-7 human breast cancer nor A549 lung cancer cell extract.

Results of this study could establish a foundation for subsequent investigation among a broad range of parasites and different types of cancer. Systematic exploration, identification and characterization of parasite/cancer shared antigens could illuminate new pathways for the potential discovery of a novel class of innovative cancer vaccine candidates and therapeutic antibodies of parasitic origin for cancer immunotherapy and targeted therapy.

## Data Availability

No datasets were generated or analyzed during the current study.

## Abbreviations

ADCC: Antibody-dependent cellular cytotoxicity
A*Sm*A: Autoclaved *S. mansoni* cercarial antigen
A*Tg*A: Autoclaved *T. gondii* tachyzoites antigen
A*Ts*A: Autoclaved *T. spiralis* larval antigen
*B. jellisoni*: *Besnoitia jellisoni*
*C. sinensis*: *Clonorchis sinensis*
*E. granulosus*: *Echinococcus granulosus*
FDA: Food and drug administration
FCA: Freund's complete adjuvant
HSP: Heat shock protein
HER-2: Human epidermal growth factor receptor-2
LL: Lewis lung
*N. caninum*: *Neospora caninum*
NF-κB: Nuclear factor kappa B
*O. viverrini*: *Opisthorchis viverrini*
PBS: Phosphate buffer saline
RIPA: Radioimmunoprecipitation assay buffer
*S. equina*: *Setaria equina*
*S. haematobium*: *Schistosoma haematobium*
*S. mansoni*: *Schistosoma mansoni*
SDS-PAGE: Sodium dodecyl polyacrylamide gel electrophoresis
STn: Sialyl-Tn
Spp: Species
TF: Thomsen Friedenreich
Tk: Thymidine kinase
Tn: N-acetylgalactosamine O-serine/threonine
*T. cruzi*: *Trypanosoma cruzi*
*T. spiralis*: *Trichinella spiralis*
*T. gondii*: *Toxoplasma gondii*
TAAs: Tumor-associated antigens
TSAs: Tumor-specific antigens
WHO: World Health Organization
